# Remarks on Lycopus Virginicus, Prinos Verticillatus, and Epiphegus Virginianus

**Published:** 1860-01

**Authors:** Charles A. Lee


*Remarks on Lycopus Virginicus, Prinos Verticillatus, and Epiphegus Virginianus.* By Charles A. Lee, M. D.

Lycopus Virginicus, (*Bugle Weed: Water Hoarhound.*)—The natural order, *Labiatæ*, to which this plant belongs, includes a large number of plants, which have been employed, from a very remote period, as aromatic cordials and stimulants. Some of them are still retained, though many have been abandoned in modern practice. They all owe their activity to volatile oil, bitter extractive, and astringent matter. The volatile oil is found in small receptacles, or globular glands, contained in the leaves, in the form of an *oleo-resin.* The *bitter extractive* is found in all the Labiatæ, and to this principle they owe their bitterness. If we add a ferruginous salt to an infusion of some of the Labiatæ, a green color is struck, which indicates the presence of *astringent matter.* Their aromatic, carminative and stimulant properties are owing to *volatile oil*; their tonic and stomachic, to *bitter extractive*, or a peculiar bitter principle. The small quantity of tannic or gallic acid which they contain only serves to increase their tonic properties. Some of them are employed in perfumery, some in cookery; while others are used in medicine, to relieve nausea and colicky pains, expel wind, prevent or relieve griping, and cover the taste of unpleasant remedies. Although volatile oil is the predominate proximate principle in the plants of this order, yet some of them contain so large a quantity of bitter extractive as to render them highly valuable as stomachics and tonics; others possess peculiar, specific properties, adapting them to fulfil certain special indications. Among this latter class may be ranked the Lycopus Virginicus.

The European species has long been celebrated as a powerful febrifuge and astringent, well adapted to the treatment of fevers and hemorrhages, while the American species has but recently been introduced into practice. The bugle weed is a common, well-known plant, growing in shady and wet places, in most parts of the United States—flowering in August—and is often confounded with the *Lycopus Sinuatus*, or water hoarhound, whose medicinal properties, though similar, are far inferior to those of the Lycopus Virginicus. The whole plant is officinal, and has a peculiar, aromatic odor, and a disagreeable bitter taste.

*Chemical Composition.*—Although the bugle weed is officinal, occupying a place in the secondary list of the United States Pharmacopœia, its chemical composition had not been accurately ascertained until the recent analysis in your own laboratory. This shows that, in seven thousand parts, it contains—

| | |
|---|---|
| Of inorganic matter, | 128 |
| Of organic matter, | 6872 |
| Total, | 7000 |

| | |
|---|---|
| Gum and albumen, | 248 |
| Tannin, | 40 |
| Bitter principle, soluble in ether, | 25 |
| Particular bitter principle, insoluble in ether, | 696 |
| Sugar, | 120 |
| Extractive, | 232 |
| Starch, | 172 |
| Clorophylle, | 220 |
| Soluble Salts, | 26 |
| Insoluble Salts, | 102 |
| Lignin, | 5120 |
| | 7000 |

The large amount of bitter principle contained in the plant is worthy of particular note, viz: seven hundred and twenty parts in seven thousand, or more than ten per cent; while the amount of tannin is inconsiderable. It contains no gallic acid.

*Therapeutical Properties and Uses.*—From the large proportion of bitter and astringent matter we might safely infer its tonic properties; but, in addition to its tonic astringent power, it possesses a narcotic virtue, though not of an active kind. The peculiar alkaloid, or oleo resinoid principle, to which it probably owes its tonic qualities, has not as yet been separated in an isolated form; the *lycopin* of some manufacturers being a powdered extract mixed with salt and other impurities. The lycopus, in certain pathological conditions, is a very valuable sedative astringent, especially adapted to cases of hemorrhage attended with frequent pulse and great nervous irritability. In such cases it often seems to prove specific, acting promptly and with great certainty in allaying irritability, while it controls the hemorrhage. It evidently strikes at the pathological cause, removing or correcting that morbid condition of the vascular and nervous system on which the hemorrhage depends; while it increases the tonicity and contractility of the minute capillaries, it diminishes the *vis-atergo*, by which the blood is propelled into them. The wild cherry bark possesses similar properties, though less strongly marked. We have used the lycopus successfully for many years, in hæmoptysis, hematemesis, mennorhagia, etc., sometimes alone, at others in conjunction with other remedies; and we have come to regard it, in certain cases, almost in the light of a specific. We are inclined to consider it best adapted to cases of bleeding from the lungs, though some practitioners regard it as most efficacious in hemorrhage from the stomach. It has been known to arrest epistaxis, when all other remedies have failed. Certainly, as a popular remedy in spitting of blood, there is no indigenous production that ranks so highly as this. Its great power, as already stated, is doubtless owing to its sedative influence over the circulatory and nervous system, while, at the same time, it constringes the smaller vessels. The late Prof. Rafinesque, whose knowledge of our indigenous botany was very accurate and extensive, remarks as follows:—"I consider the bugle weed a very good substitute for all narcotics, prussic acid, and even bleeding, since it produces the same state of the pulse and arterial system, without inducing any debility, or acting on the heart and brain in any injurious manner." While we do not admit that any vegetable remedy is a perfect substitute for blood-letting, in all cases, it must nevertheless be conceded that the bugle weed will moderate the force and frequency of the pulse, and thus accomplish one of the important indications of bleeding, unattended with the danger of relaxing the minute vessels—the source of the hemorrhage. We have called the lycopus a *tonic*, though its tonic properties are not strongly marked. In this respect it yields to the cerasus ; it checks the secretions like most astringents, while it quiets the circulation and allays inordinate irritability. These properties render it useful in most cases of excessive flux, associated with such a condition. Besides the various forms of hemorrhage above mentioned, it will be found well adapted to many cases of diabetes, senile cough, humoral asthma, chronic diarrhœa, etc. In the latter, when caused by irritation, it proves particularly serviceable, after thorough evacuation by castor oil. The European species has been found very efficacious as a remedy for intermittents, given in powder previous to the access, and it is very probable that our own spices possesses similar properties. It seems to have been used from time immemorial, as it is mentioned in the most ancient records. It forms a very good black dye, and Withering says that gipsies stain their skin with it.

The physiological effects of the bugle weed are such as might be inferred from what has been already stated in regard to its therapeutical effects. Taken in health, in the form of a strong infusion, in doses of a wine-glass full every two hours, it abates the force and frequency of the pulse, without nausea or cerebral disturbance, while at the same time it causes slight constipation.

*Preparations.*—Infusion, decoction, fluid extract, syrup, tincture. The infusion, made by pouring a pint of boiling water to an ounce of the dried plant, is the most frequent form of administration. Of this, in hæmoptysis, a wine-glass full should be given as often, at first, as every half-hour or hour, according to the urgency of the symptoms. The *fluid extract* from your establishment has proved a reliable preparation, in doses of from one to two drams every two hours. A good extemporaneous infusion is made with one ounce of the fluid extract to one pint of water ; dose, two to four ounces. The syrup may be prepared from the infusion, or by mixing three ounces of the fluid extract with twelve ounces of simple syrup; dose, one to two ounces.—*Journal of Materia Medica.*

